# ﻿Mammal diversity survey of Ko Pha-ngan in Surat Thani Province, Thailand

**DOI:** 10.3897/zookeys.1229.118127

**Published:** 2025-02-25

**Authors:** Dawn R. Cook-Price, Olga N. Petko, Sunchai Makchai, Taksin Artchawakom, Pongthep Suwanwaree

**Affiliations:** 1 School of Biology, Institute of Science, Suranaree University of Technology, Nakhon Ratchasima, 30000, Thailand Suranaree University of Technology Nakhon Ratchasima Thailand; 2 Naja Project Ko Pha-ngan, Ko Pha-ngan, Surat Thani, 84280, Thailand Naja Project Ko Pha-ngan Ko Pha-ngan Thailand; 3 Thailand Natural History Museum, National Science Museum, Pathum Thani, 12120, Thailand Thailand Natural History Museum, National Science Museum Pathum Thani Thailand; 4 Population and Community Development Association, Saptai 98 Moo 6 Phaya Yen, Pak Chong District, Nakhon Ratchasima, 30320, Thailand Population and Community Development Association Nakhon Ratchasima Thailand

**Keywords:** Biodiversity, conservation, insular populations, island biogeography, species list

## Abstract

This study aims to survey mammal diversity on Ko Pha-ngan, located 80 km off the east peninsular coast of Surat Thani province, Thailand. Thirteen camera trap sites, 32 transects, six drift line fence traps, five mist net trap sites, and nine live trap sites placed in human settlement, human-disturbed forest, and national park forest from February 2021 to September 2023 were utilized. A total of 28 mammal species of eight orders, 17 families, and 21 genera were found. Among them, 11 species are flying mammals while the remaining are terrestrial. Of the species detected, *Manisjavanica* (pangolin) is critically endangered, while *Nycticebuscoucang* (slow loris monkey) is endange by IUCN Red List. Additionally, *Rusaunicolor* (sambar deer) is vulnerable and Pteropuscf.hypomelanus (island flying fox), *Trachypithecusobscurus* (dusky leaf monkey), and *Ratufabicolor* (giant black squirrel) are near threatened. These findings highlight the need to conserve and protect both national park forest and human-disturbed forest from anthropogenic pressures due to the finite area of an island, in which potential local extinction risk is higher.

## ﻿Introduction

World mammal populations are at risk. Global conservation efforts are often approached after a threat has occurred and scientists scramble to rectify damage that has already taken its toll (Cardillo et al. 2023). The number of mammal species is continually fluctuating (new species discovered and existing species extinction) and depending on sources, the count ranges from 5,400 (Wilson and Reeder 2005) to more than 6,700 in the Mammal Diversity Database (2023). Of the global mammal population, Thailand’s biodiverse realm shelters approximately 302 mammalian species (Office of Natural Resources and Environmental Policy and Planning 2017). Among these, 136 face threats ranging from near-threatened status to critically endangered.

Ko Pha-ngan, 80 km west of the mainland Surat Thani Province, is largely under the protection of the Than Sadet-Ko Pha-ngan National Park. Its ecological diversity stands out and has not been fully investigated. While Thailand boasts a commendable national park system, information about the species on the island of Pha-ngan is often gleaned from antiquated records or unverified crowd-sourced platforms. The national park species list consists of a mere eight mammal species (Department of National Parks, Wildlife and Plant Conservation 2018), a figure this study seeks to revise and expand.

Ko Pha-ngan’s insular ecosystem, characterized by its confined area and distinct biosphere, is particularly sensitive to both natural and anthropogenic changes. Home to the infamous Full Moon Party (Malam 2008), Ko Pha-ngan is known for its vibrant festivities and role as a major tourist attraction but it faces significant ecological challenges. Historically, Ko Pha-ngan has experienced extensive transformations: from the tin mining of the 1970s, the emergence of coconut plantations, and the contemporary party scene (Nutalaya et al. 1979). Human-induced disturbances, paired with a tourism boom that attracted more than one million visitors in 2017 (Kaewcharoen et al. 2019) have precipitated rapid deforestation and infrastructure development. Such changes have encroached upon natural habitats and may have consequential impacts on the island’s fauna as, globally, islands are known to house 15% of global mammal, bird, and amphibian populations (Deidun 2011; Berger-Tal and Saltz 2019).

This research was designed to catalog the mammalian species of Ko Pha-ngan through a comprehensive survey using camera traps, foot surveys, and trapping. The study enhances our understanding of species distribution and abundance across the island’s various habitats. By leveraging a multifaceted approach, this work lays the groundwork for future ecological studies and conservation strategies and policies, crucial for safeguarding the island’s mammalian diversity in the face of ongoing environmental challenges.

## ﻿Methods

**Figure 1.**
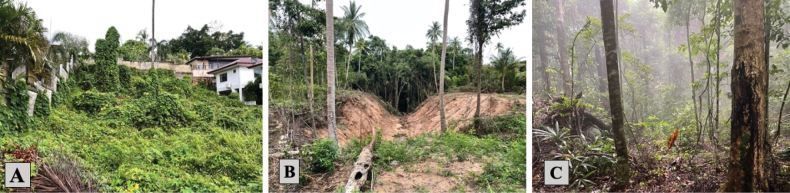
**A** human settlement **B** human-disturbed forest **C** national park forest.

Ko Pha-ngan (latitude 9.74939, longitude 100.02649), dominated by the Than Sadet-Ko Pha-ngan National Park covers an expanse of 125 km^2^ (Department of National Parks, Wildlife and Plant Conservation 2018). The island’s flatter areas are predominantly residential (resorts as well as other single-family residents) and agricultural (coconut plantations, rubber trees, small dragon fruit plots, and durian farms) interspersed throughout the residential infrastructure and forested areas. In contrast, montane forests in higher elevated regions consist of a diverse range of flora and fauna including dipterocarps and various ferns while providing critical habitat for a range of wildlife including reptiles, amphibians, and mammals (Koh Phangan City 2023). From February 2021 to September 2023, a comprehensive mammalian survey spanning both rainy and dry seasons was conducted on the island including human settlement (**HS**), human-disturbed forest (**HDF**), and national park forest (**NPF**, Fig. 1).

**Figure 2.**
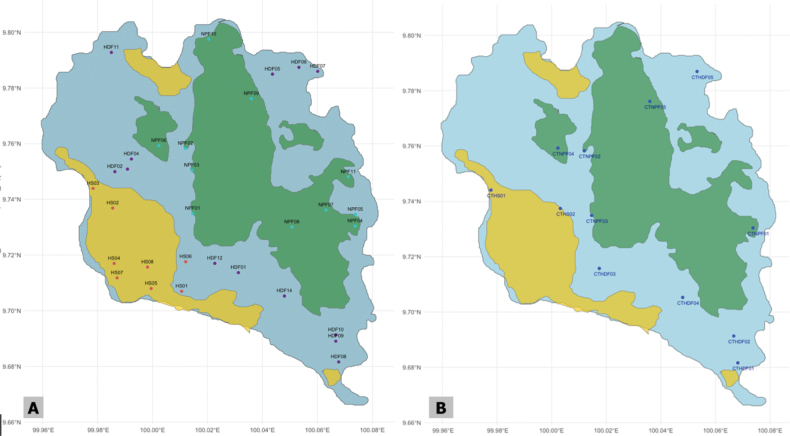
**A** transect locations and **B** camera trap locations in human settlement (yellow), human-disturbed forest (blue), and national park forest (green).

**Figure 3.**
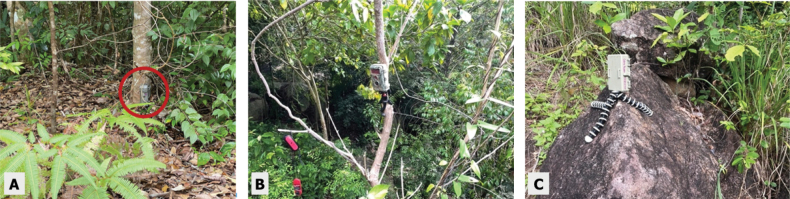
**A** camera trap positioned on a tree **B** facing the ground **C** on a rock.

Camera trapping, a passive and low-cost strategy (Djekda et al. 2020) was implemented across 13 locations on the island: three in HS, five in HDF, and five in NPF (Fig. 2A). Locations were carefully selected based on several key criteria: proximity to water sources, evidence of mammal activity (such as tracks, scat, or sightings), and ease of access for regular monitoring. Water is crucial for mammals, especially during the dry season, making these areas ideal for increased potential mammal activity. In addition, signs of mammal activity guided placement in hope of capitalizing on potential detection. Due to the island’s challenging topography, particularly in the montane regions, some sites were chosen based on the practical limitations of accessibility with the aim of achieving a representative sample across habitat types while ensuring safety for researchers and equipment alike. Camera traps were installed in clusters of three at each site (total of 39 cameras), maximizing the detection scope as cameras placed at different angles can increase detection potential of species (Fig. 3). Cameras were used in this manner to monitor sites with known mammal activity in addition to sites chosen to better understand mammal potential in an area (Burton et al. 2015). Camera settings varied as two different cameras were used for this study. The BlazeVideo A252 trail camera (BlazeVideo, UK) was used in conjunction with the Stealth Cam STC-G42NG (Stealth Cam, Texas, USA). Settings for mammals varied utilizing a burst of three photos or a burst of three photos as well as a 15-second video. Deployment spanned a total of 730 days, with individual cameras operating between 14 to 180 consecutive days. All photographs were examined and each individual in the frame was counted. If a burst of three photos had an individual in all three photos the species was only counted once as it was the same individual. If time lapsed, then the individual was counted again as a new sighting; however, we recognize the limitations this method has and acknowledge there is no accurate way to account for multiple individuals or the same individual multiple times.

Parallel to camera trapping, a series of 78 visual surveys were conducted bi-weekly, primarily targeting species that might evade camera traps such as the dusky leaf monkey, slow loris, and giant black squirrel, which are primarily arboreal and out of the scope of camera trap placement. Teams of up to seven individuals participated in these surveys, encompassing 1,343 hours predominantly conducted during nighttime. The survey utilized 32 transects across all three habitats (Fig. 2B), with lengths dictated by the terrain’s navigability varying from 500 m to 5 km and elevation spanning from sea level to 627 m. For mapping and analysis purposes, one geographic coordinate was recorded for each survey site, irrespective of the transect length. Each species was counted during the survey and totaled at the end of the survey. Individuals were only counted when surveyors moved in from the start of a transect (inward leg). No species was counted on the way out (outward leg) from the survey transect to avoid double counting.

**Figure 4.**
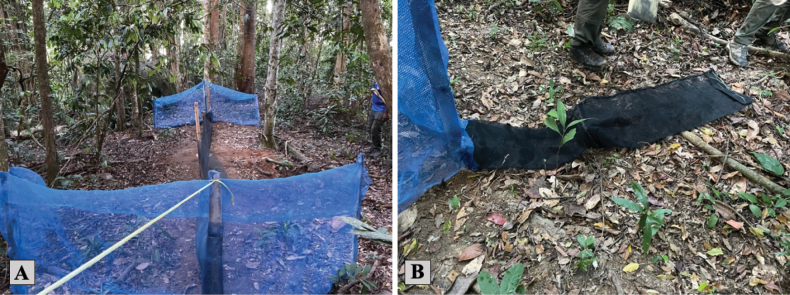
**A** complete view of a drift line fence **B** funnel trap.

Additionally, six specialized drift line fence traps were installed from February 2022 to September 2023, with daily checks for a total of 236 days. Traps remained open ranging from 7 to 90 days and checked daily. These traps featured a dual funnel design with a central pitfall component, strategically complemented by camera traps to enhance species capture rates (Fig. 4).

Nine small mammal trap sites were set in conjunction with other trapping methods (camera, funnel trap, and pitfall trap) for better identification of small mammal species. Small mammal traps were placed approximately 10 m from the drift line fence as well as 10 m from each trap in a three-trap array. Traps are 310 mm long × 150 mm wide × 335 mm high and appropriate for a range of small mammals including rats, shrews, and squirrels. Bait consisted of banana, peanut butter, carrot, and various breads. Traps were left open for a minimum of 5 days and a maximum of 45 days through the study period.

**Figure 5.**
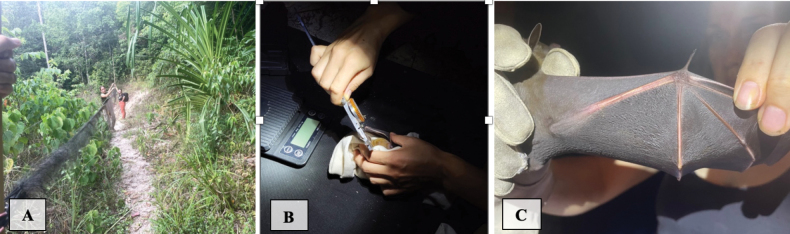
**A** setting up a mist net **B** measuring bat ear length and **C** bat wing splayed for measurement of humorous bone.

To better identify the bat species seen on camera traps and foot surveys, we utilized mist nets (Fig. 5) in five different locations throughout the island. Two in the national park forest, two in the human-disturbed forest habitat, and one in the human settlement area including a bamboo patch within it. The nylon mist net (10 m × 2 m) was placed across trails, near streams, and in areas near edge habitats. Nets were set up just before dusk and remained so for approximately 4–8 hours. Nets were continuously monitored, and bats were immediately untangled for the collection of morphometric data. Photographs and measurements were taken for a more accurate differentiation between species (Fig. 5).

In conducting our research, species identification was rigorously performed using a variety of reliable sources. The American Society of Mammologists’ Mammal Diversity Database (2023) and the database of Thailand’s Natural History Museum (2023) http://nhmsearch.nsm.or.th, were primary references. For the taxonomy of Murinae rodents, we relied on the detailed keys provided by Pimsai et al. (2014). The complexities of the *Tupaia* species were addressed with the guidance of Sargis et al. (2013). The books by Lekagul and McNeely (1988), and Francis (2008) were indispensable for broader mammalian identification within Thailand. For the specific identification of bat species, the field survey by Duenkae (2010) offered comprehensive insights.

## ﻿Results

The survey identified 28 mammalian species across 16 families, contributing 19 new species records to Ko Pha-ngan’s mammal diversity list (Table 1). This includes 11 species of Chiroptera from seven genera, seven Rodentia species from four genera, three primate species, two species each of Artiodactyla and Carnivora, and one species each of Pholidota, Scandentia, and Soricomorpha. Notable among these findings are species with significant conservation statuses according to the IUCN Red List (2024) and the Thai Red List of threatened species. *Manisjavanica* (pangolin) is listed as critically endangered (CR) by IUCN and endangered (EN) on the Thai Red List (Nabhitabhata 2005). *Nycticebuscoucang* (slow loris) is classified as endangered (EN) by IUCN and vulnerable (VU) in Thailand. In addition, *Rusaunicolor* (sambar deer) is listed as vulnerable (VU) on both lists. Three additional species, Pteropuscf.hypomelanus, *Trachypithecusobscurus*, and *Ratufabicolor*, are categorized as Near Threatened (NT) by IUCN while the Thai list ranks them as Vulnerable, Vulnerable and Least Concern, respectively. The most vulnerable species, *Manisjavanica*, *Nycticebuscoucang*, *Rusaunicolor*, and *Ratufabicolor*, were primarily found in the national park forested areas; however, *Nycticebuscoucang* and *Ratufabicolor* were occasionally seen on the edges of human-disturbed forests. These species are protected under the Convention on International Trade in Endangered Species of Wild Fauna and Flora (CITES), which lists them under Article I and Article II of the treaty. Additionally, to combat illegal trafficking, Thailand has implemented the Wildlife Conservation and Protection Act (2019), which enhances legal protection for threatened species and implements fines for violations involving protected wildlife.

**Table 1.** Mammal species list on Ko Pha-ngan, IUCN conservation status, Thailand Red List of threatened species, detection method, and habitat.

Order	Family	Species	IUCN Status	Thailand Red List	Detection Method	Habitat		
						HS	HDF	NPF
Carnivora	Viverridae	Paradoxurusmusangus*	LC		CT		x	x
	Herpestidae	Herpestesjavanicus*	LC	LC	CT			x
Artiodactyla	Cervidae	*Rusaunicolor*	VU	VU	CT		x	x
	Suidae	Susscrofa*	LC	LC	CT		x	x
Rodentia	Sciuridae	*Callosciuruscaniceps*	LC	LC	CT, LT, S	x	x	x
		Callosciuruserythraeus*	LC	LC	CT, LT, S	x	x	x
		Ratufabicolor*	NT	LC	OP		x	x
	Muridae	*Maxomyssurifer*	LC	LC	CT, LT, S		x	x
		Rattusandamanensis*	LC	VU	CT, LT, S		x	x
		Rattusexulans*	LC	LC	LT	x		
		Rattustanezumi*	LC	LC	CT, LT, S		x	x
Soricomorpha	Soricidae	Crocidurafuliginosa*	DD	LC	LT, S		x	x
Scandentia	Tupaiidae	*Tupaiabelangeri* *	LC	LC	CT, LT		x	x
Primates	Cercopithecidae	Macacafascicularis*	LC	LC	CT, OP, S	x	x	x
		Trachypithecusobscurus*	NT	VU	OP			x
	Lorisidae	Nycticebuscoucang*	EN	VU	S		x	x
Pholidota	Manidae	Manisjavanica*	CR	EN	CT, S			x
Chiroptera	Rhinolophidae	Rhinolophuscf.affinis*	LC	LC	MN	x	x	x
		Rhinolophuscf.pusillus*	LC	LC	MN	x	x	x
	Hipposideridae	Hipposideroslarvatus*	LC	LC	MN	x	x	x
		Hipposiderosarmiger*	LC	LC	MN	x	x	x
	Vespertilionidae	Myotiscf.horsfieldii*	LC	LC	MN	x	x	x
		*Tylonycterismalayana*	DD	DD	MN	x		
	Pteropodidae	Pteropuscf.hypomelanus*	NT	VU	MN	x	x	x
		Cynopterushorsfieldii*	LC	LC	MN	x	x	x
		Cynopterussphinx*	LC	LC	MN	x	x	x
		Eonycterisspelaea*	LC	LC	MN	x	x	x
	Megadermatida	Megadermaspasma*	LC	LC	MN	x	x	x

HS = human settlement, HDF = human-disturbed forest, NPF = national park forest, LC = least concern, VU = vulnerable, NT = near threatened, EN = endangered, CR = critically endangered, CT = camera trap, LT = live trap, OP = Opportunistic, S = survey, MN = mist net. *Indicates new record.

**Figure 6.**
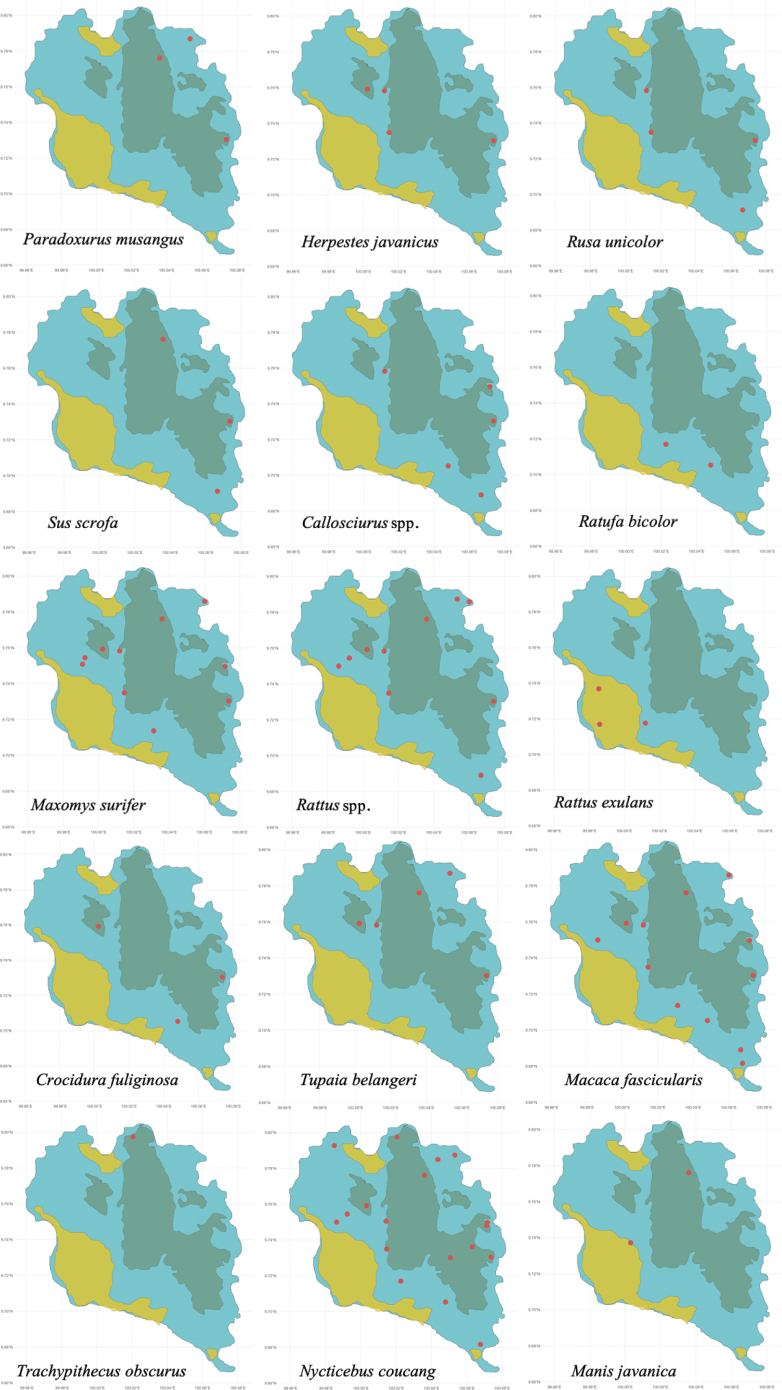
Detection for each species by habitat (green = NPF, blue = HDF, and yellow = HS).

Of the 28 species detected (Table 1), 25 were observed in national park forest areas (11 flying and 15 non-flying), 19 were observed in human-disturbed forest (11 flying and 9 non-flying), and 12 were observed in human settlement areas (11 flying and 2 non-flying) (Fig. 6). Notably, all flying mammal (bat) species were present in all three habitats likely due to their flight range, while *Rattusexulans* was restricted to human settlement areas. *Nycticebuscoucang* and *Ratufabicolor*, species of conservation concern, were primarily observed in national park and human-disturbed forests. Three species were detected exclusively in national park forest (*Herpestesjavanicus*, *Trachypithecusobscurus*, and *Manisjavanica*). Only one non-flying species, *Macacafascicularis*, was detected in all habitat types. These findings suggest that while some species exhibit considerable adaptability to human-altered environments, others, particularly those listed as endangered or vulnerable, show a marked preference for less disturbed habitats.

All bat species were detected in each of the habitats which was expected due to their flight range; however, they were only confirmed via mist net trapping despite detection on camera traps and on night surveys. This is due to the difficulty of differentiating species in a black and white photograph or a fly-by. Similarly, rodent species were frequently observed in both human-disturbed forest and national park forest during night surveys; however, species identification was only made from live traps where a more comprehensive assessment of identifying features could be made. During night surveys, detection was noted for rodents as a whole category (e.g., rats, squirrels, and shrews).

Of the detection methods 12 species were detected and identified via camera trap (Table 1). Four of these were exclusively camera-trap detections with three species (*Tupaiabelangeri*, *Macacafascicularis*, and *Manisjavanica*) additionally detected opportunistically, on survey and in live traps. One mammal, *Macacafascicularis*, was detected via all detection methods. The slow loris (*Nycticebuscoucang*) was exclusively detected via surveys (Table 2, Fig. 14). Due to their primarily arboreal nature, they were not detected on camera traps; most opportunistic encounters occurred during the day and being nocturnal, minimized detectability. Live traps lead to the identification and detection of eight species consisting of rodents and shrews. Flying mammals and rodents were detected via camera trap; however, it was not possible to obtain an accurate identification. The observation was counted as a general animal group sighting rather than species.

**Table 2.** Number of species detected in each habitat and method of detection.

Habitat / Detection method	HS				HDF				NPF				Total
	CT	OP	LT	S	CT	OP	LT	S	CT	OP	LT	S	
*Paradoxurusmusangus*					1				5				6
*Herpestesjavanicus*									6				6
*Rusaunicolor*					4				7	1			12
*Susscrofa*					5				8				13
*Callosciurus* spp.					2	2	1		3	2	2		12
*Ratufabicolor*						1				2			3
*Maxomyssurifer*							3	8	9		5		25
*Rattusexulans*			4										4
*Rattus* spp.							4	13	16		7		40
*Crocidurafuliginosa*								1			7		8
*Tupaiabelangeri*							1		8		2		11
*Macacafascicularis*		6				9		8	18				41
*Trachypithecusobscurus*										1			1
*Nycticebuscoucang*								11				21	32
*Manisjavanica*									1			1	2
Total		6	4		12	13	9	41	81	6	23	22	216

HS = human settlement, HDF = human-disturbed forest, NPF = national park forest, CT = camera trap, LT = live trap, OP = opportunistic, S = survey, and MN = mist net.

Due to the nature of this study, we cannot differentiate individuals within a species which is the reason for using only presence/absence data. For instance, Sambar deer (*Rusaunicolor*) were detected via camera trap on multiple occasions without knowing if it was two individuals (one male and one female) detected multiple times or multiple individuals.

The most detected species on the island was *Macacafascicularis* (macaque) with 41 sightings. They were detected via all detection methods (Table 2). Numbers are likely higher due to the way the species generally stays in a troop and can often be seen with others. Closely following the macaques in detection was *Nycticebuscoucang* (slow loris monkey). Though the slow loris is a solitary, arboreal and nocturnal creature, the surveys at night allowed us to spot their distinct eyeshine easily. The least detected species was *Trachypithecusobscurus* (dusky leaf monkey) that was only detected opportunistically on one occasion. This is likely due to their shy nature and use of the high canopy. Rats and flying mammals are excluded as they were numerous; however, several of these mammals were easy to detect without differentiation between individual species when running or flying by on a survey.

### ﻿Class Mammalia

**Order Carnivora**

**Family Viverridae**

#### ﻿ Paradoxurus musangus

Taxon classificationAnimaliaCarnivoraViverridae

(Raffles, 1821)

0ABBC874-4DD8-5B34-AA6D-5CF12FB985D6

Fig. 7A Palm Civet

##### Notes.

This species was detected on six occasions (Table 2), five times in national park forest areas in two different camera trap sites and one camera trap site in human-disturbed forest area (Fig. 6). Nocturnal species detected exclusively via camera trap in remote forested areas, the palm civet’s diet includes insects, fruits, and small mammals. Its presence highlights the intactness of nocturnal food webs within the national park forest habitat (Veron et al. 2015).

**Figure 7.**
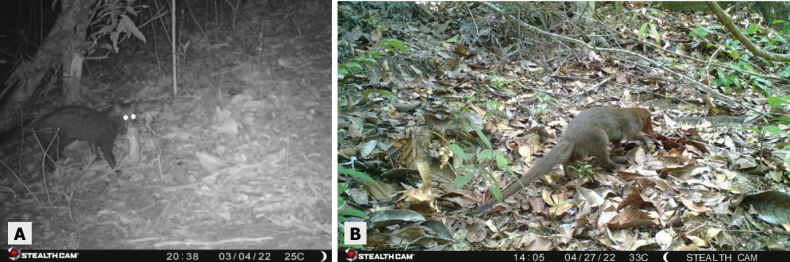
**A** Civet with prey item in national park forest habitat **B** Mongoose near trap site in national park forest habitat.

### ﻿Family Herpestidae

#### ﻿ Herpestes javanicus

Taxon classificationAnimaliaCarnivoraHerpestidae

(Saint-Hilaire, 1818)

2575EBB5-0D8D-5D0D-80F9-48070B85CC8A

Fig. 7B Javan mongoose

##### Notes.

This species was detected on camera traps on five different occasions (Table 2) in two different areas of national park forest transects (Fig. 6). Diurnally (daytime) actively foraging for mainly insects; however, also known to eat snakes, frogs, small mammals, and birds (Wilson and Mittermeier 2009).

### ﻿Order Artiodactyla

**Family Cervidae**

#### ﻿ Rusa unicolor

Taxon classificationAnimaliaArtiodactylaCervidae

(Kerr, 1792)

DCF8712A-0CCA-5B73-8699-3AEFA5EB5741

Fig. 8 Sambar Deer

##### Notes.

This species is nocturnal and herbivorous feeding on a variety of plants including trees, shrubs, and grasses found in the forested areas they have been observed (Suksawat et al. 2018). The sambar deer has been detected twelve times (Table 2) via camera trap in eight transects in national park forest and three different human-disturbed forest transects connected to national park forest; however, there was one opportunistic observation directly across the stream from the headquarters of the national park.

**Figure 8.**
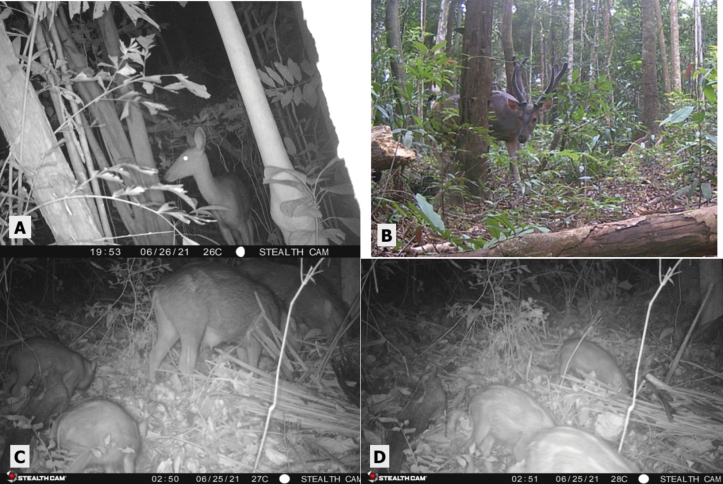
**A** female Sambar deer in national park forest habitat **B** male Sambar deer in national park forest habitat **C** wild boar family in human-disturbed forest habitat, and **D** three wild boar piglets in human-disturbed forest habitat.

### ﻿Family Suidae

#### ﻿ Sus scrofa

Taxon classificationAnimaliaArtiodactylaSuidae

(Linnaeus, 1758)

EF1C638C-580F-5909-A7E3-63588F597809

Fig. 8 Wild boar

##### Notes.

This species was observed thirteen times on camera traps (Table 2). It was observed in two national park forest transects and one human-disturbed forest transect (Fig. 6). The first observation of three piglets were sighted with an adult female, presumably the mother (enlarged teats) and a few months later in a different camera trap area (further north), an adult and larger sized piglets were observed. These wild boars can inflict a large amount of damage to plants in an ecosystem (Ickes 2001).

### ﻿Order Rodentia

**Family Sciuridae**

#### ﻿ Callosciurus erythraeus

Taxon classificationAnimaliaRodentiaSciuridae

(Pallas, 1779)

2F9B517E-A5FE-52D8-BB6D-41AA3E6354FC

##### Notes.

This species was occasionally observed in coastal forested areas in an opportunistic manner. It was captured once in live traps (Table 2), and in five different camera trap locations in central forested areas of both national park and human-disturbed forest (Fig. 6).

#### ﻿ Callosciurus caniceps

Taxon classificationAnimaliaRodentiaSciuridae

(Grey, 1842)

32EF31B6-22A1-563D-80E7-F8FC397114A4

##### Notes.

This species was observed in coastal forested areas occasionally in an opportunistic manner and twice in live traps (Table 2) at two different sites in national park forest, however, more often on camera traps at five different trap sites in central forested areas of both national park and human-disturbed forest.

#### ﻿ Ratufa bicolor

Taxon classificationAnimaliaRodentiaSciuridae

(Sparrman, 1778)

F0B57C4D-AF7E-502F-9829-CE9C34613648

**Figure 9.**
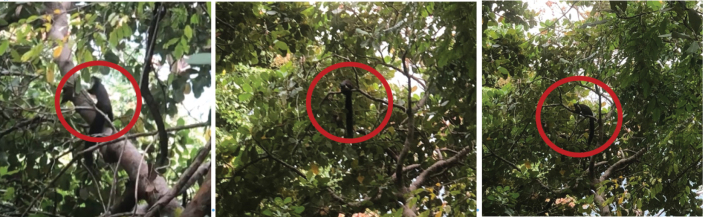
*Ratufabicolor* (giant black squirrel) opportunistically observed in human-disturbed forest habitat.

Fig. 9 Giant black squirrel

##### Notes.

Three individuals of this species were sighted opportunistically (Table 2) in two different human-disturbed forest areas (Fig. 6). Herbivorous and arboreal, this species eats leaves, various fruits, and seeds (Tongkok et al. 2020). Each occasion this species was sighted was in secluded forest areas between human settlement areas.

### ﻿Family Muridae

#### ﻿ Maxomys surifer

Taxon classificationAnimaliaRodentiaMuridae

(Miller, 1900)

B8D7FEC9-8884-5369-BAD0-51F8BEADE51B

**Figure 10.**
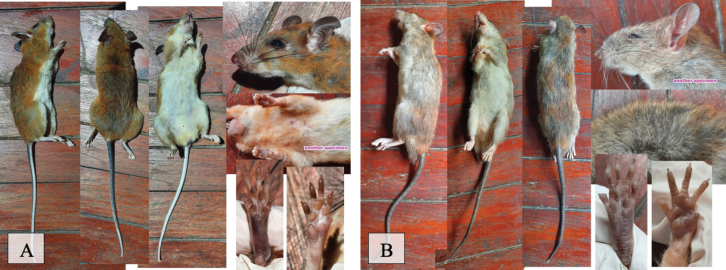
**A***Maxomyssurifer* (red spiny rat) **B***Rattusandamanensis* (Sikkim rat).

Fig. 10A Red spiny rat

##### Notes.

Twenty-five individuals (Table 2) of this species were captured on camera trap in the national park forested areas and in live traps in three different national park forest transects and two human-disturbed forest transect areas (Fig. 6). Nocturnal and omnivorous feeding on both insects and various plant matter primarily in the forest (Tongkok et al. 2020).

#### ﻿ Rattus andamanensis

Taxon classificationAnimaliaRodentiaMuridae

(Blyth, 1860)

54E13F0D-5681-55FD-969B-90C7DA11AB1A

Fig. 10B Indochinese Forest Rat/Sikkim rat

##### Notes.

This nocturnal and omnivorous species was captured in live traps (twice in human-disturbed forest trap sites and three times in national park forest trap sites) and on camera traps (Table 2) in five human-disturbed forest transects and five national park forest transects (Fig. 6).

#### ﻿ Rattus exulans

Taxon classificationAnimaliaRodentiaMuridae

(Peale, 1848)

544263D1-9F2B-557C-BE9B-28DEBFC289F2

**Figure 11.**
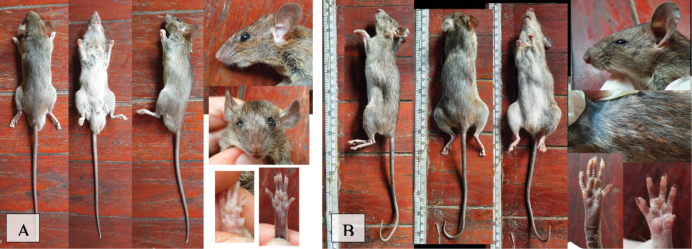
**A***Rattusexulans* (Polynesian rat) **B***Rattustanezumi* (Tanezumi rat).

Fig. 11A Polynesian Rat

##### Notes.

This nocturnal and omnivorous species was captured in a live trap in three human settlement areas, and primarily lives around homes and restaurants. The authors acknowledge the map shows one human-disturbed area; however, it was trapped at a restaurant and therefore its proximity in human settlement determined the categorization.

#### ﻿ Rattus tanezumi

Taxon classificationAnimaliaRodentiaMuridae

(Temminck, 1844)

AFDC9D79-8A2E-5BB8-AD34-1435027BC836

Fig. 11B Tanezumi rat

##### Notes.

This species was caught in live traps in addition to camera traps in five of the of the national park forested transects, and five of the human-disturbed forest transects.

### ﻿Order Soricomorpha

**Family Soricidae**

#### ﻿ Crocidura fuliginosa

Taxon classificationAnimaliaSoricomorphaSoricidae

(Blyth, 1855)

F260839D-DC7F-5DCE-BC49-FF36FD7539BC

**Figure 12.**
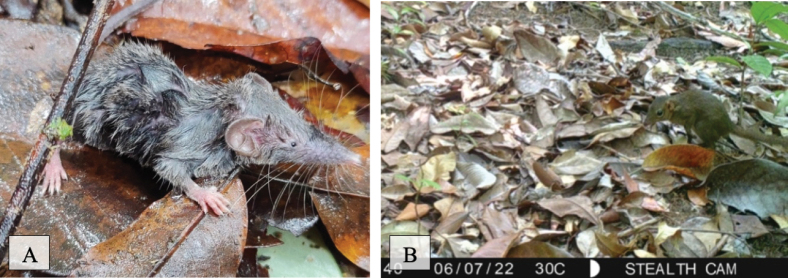
**A***Crocidurafuliginosa* (Southeast Asian shrew) **B***Tupaiabelangeri* (northern treeshrew).

Fig. 12A Southeast Asian shrew

##### Notes.

Eight individuals (Table 2) of this species were caught by funnel traps at two sites in the national park forest and observed once on a survey in human-disturbed forest. The survey sighting was on a newly disturbed dirt wall in the HDF014 transect area approximately 20 m from the entrance of an island party site called “Waterfall Party”. This was the first and only time this species was observed in a survey. The location is in a human-disturbed forest very near national park forest in the southeast central area of the island. Distinguishing features of this shrew include dark fur (black) with a silvery gloss and the tail is long and thin (Hai et al. 2020).

### ﻿Order Scandentia

**Family Tupaiidae**

#### ﻿ Tupaia belangeri

Taxon classificationAnimaliaScandentiaTupaiidae

(Wagner, 1841)

9DBD2E6F-60E0-5AE5-B908-225025A99740

Fig. 12B Common tree shrew

##### Notes.

Eleven individuals (Table 2) of this species were observed on camera traps in four national park forested transects and camera sites in addition to capture via live traps in two different national park forest sites and one human-disturbed forest site (Fig. 6).

### ﻿Order Primates

**Family Cercopithecidae**

#### ﻿ Macaca fascicularis

Taxon classificationAnimaliaPrimatesCercopithecidae

(Raffles, 1821)

D2D9AE25-9242-556B-BF53-D853CE9CE735

**Figure 13.**
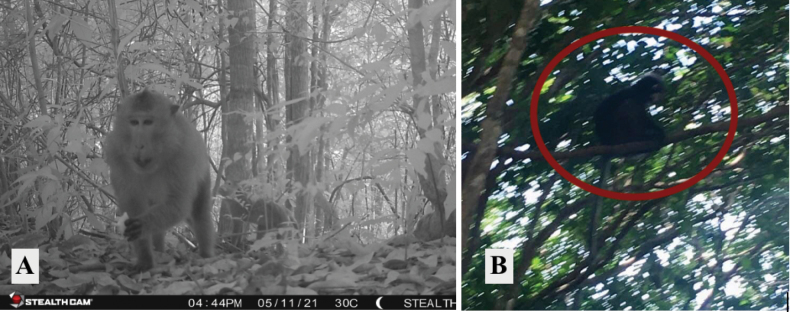
**A***Macacafascicularis* (long-tailed macaque) **B***Trachypithecusobscurus* (dusky leaf monkey).

Fig. 13A Long-tailed macaque

##### Notes.

*Macacafascicularis* was the most observed species detected via all detection methods, with 41 individuals (Table 2) were observed on foot surveys and multiple camera traps throughout both national park forest areas (6 different transects or camera sites) and human-disturbed forest areas (6 different transects or camera sites) on the island. There is one small troop that can be seen in the southeast area of the island; however, this troop stays near the road and residential areas as humans intentionally leave food items. This lone troop behaves differently than the other troops. The wild troops encountered were very well hidden in the forest areas away from human settlement areas and throughout the island (Fig. 6).

#### ﻿ Trachypithecus obscurus

Taxon classificationAnimaliaPrimatesCercopithecidae

(Reid, 1837)

8C93F8E9-2152-5CF1-B724-2AD2F383E543

Fig. 13B Dusky leaf monkey

##### Notes.

One individual was observed by an island resident in a coastal forested area on the edge of human-disturbed forest and national park forest areas. Technically the detection was on the national park forest edge and well within our transect in the national park. This species is elusive and has not been detected via camera trap or any of the other methods of detection.

### ﻿Family Lorisidae

#### ﻿ Nycticebus coucang

Taxon classificationAnimaliaPrimatesLorisidae

(Boddaert, 1785)

70CF7E5B-16FC-5A2D-B7D5-5EEACF6C38E8

**Figure 14.**
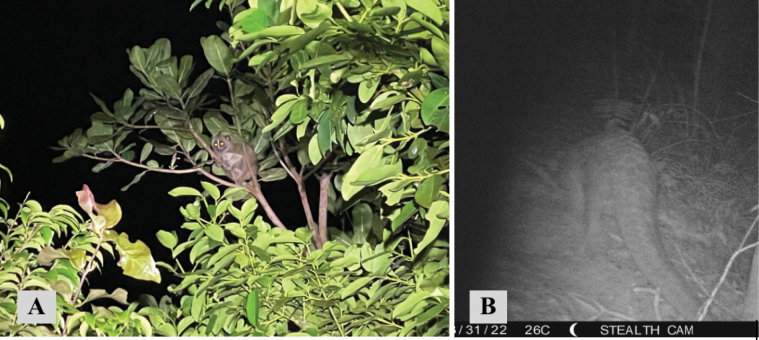
**A***Nycticebuscoucang* (Sunda slow loris) observed on the edge of human-disturbed forest path leading to national park forest **B***Manisjavanica* (Sunda pangolin) detected on a camera trap in a national park forest.

Fig. 14A Sunda slow loris

##### Notes.

Thirty-two individuals (Table 2) of this globally endangered species were observed exclusively on night surveys in eight of the 14 human-disturbed forest areas and nine of the ten national park forested areas. On one occasion this species was observed in a HDF patch very near a HS area. This species was seen throughout the island.

### ﻿Order Pholidota

**Family Manidae**

#### ﻿ Manis javanica

Taxon classificationAnimaliaPholidotaManidae

(Desmarest, 1822)

70A74094-0F32-5FD4-8C18-746BD3D26E78

Fig. 14B Sunda pangolin

##### Notes.

Two individuals of this species were observed once on a night survey in a national park forest area and once on a camera trap in a different national park forest transect.

### ﻿Order Chiroptera

**Family Rhinolophidae**

#### ﻿ Rhinolophus affinis

Taxon classificationAnimaliaChiropteraRhinolophidae

(Horsfield, 1823)

99CF8A9E-A93F-5A38-A364-7BFF74D91C9F

**Figure 15.**
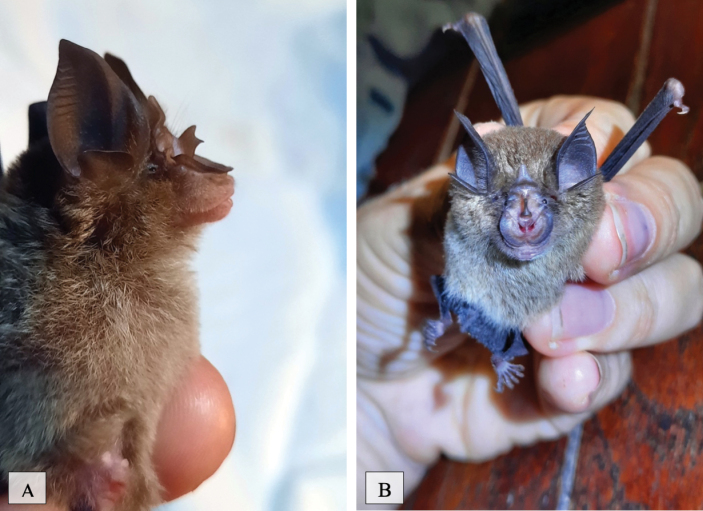
**A**Rhinolophuscf.pusillus**B***Rhinolophusaffinis*.

Fig. 15B Intermediate horseshoe bat

##### Notes.

This insectivorous species (Mickleburgh et al. 1992) was observed in human-disturbed forest areas, national park forest, and human settlement areas located near forested areas. This species was trapped in all five mist net trapping sites.

#### ﻿ Rhinolophus pusillus

Taxon classificationAnimaliaChiropteraRhinolophidae

(Temminck, 1834)

B4AF77FB-5D75-522E-8190-05F9F40EB476

Fig. 15A Least horseshoe bat

##### Notes.

This insectivorous species (Mickleburgh et al. 1992) was observed in human-disturbed forest areas, national park forest, and human settlement areas located near forested areas. This species was also trapped in all five mist net trap sites.

### ﻿Family Hipposideridae

#### ﻿ Hipposideros larvatus

Taxon classificationAnimaliaChiropteraHipposideridae

(Horsfield, 1823)

8C97EEDC-F063-5A00-B85B-3BE7B092089A

**Figure 16.**
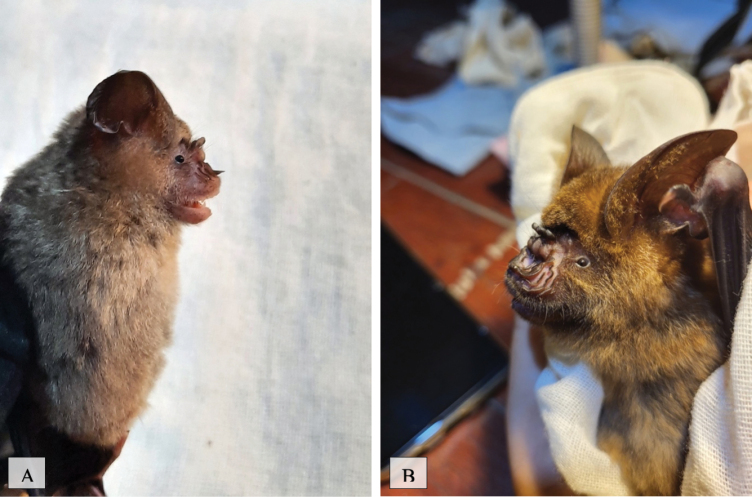
**A***Hipposideroslarvatus***B***Hipposiderosarmiger*.

Fig. 16A Intermediate roundleaf bat

##### Notes.

This species was observed in human-disturbed forest, national park forest, and human settlement areas located near forested areas. This species was trapped in all five mist net trap sites. This species primarily feeds on flying insects such as beetles and can commonly be found at forest edges (Pavey 2021).

#### ﻿ Hipposideros armiger

Taxon classificationAnimaliaChiropteraHipposideridae

(Hodgson, 1835)

FFAED57E-93CF-5B2B-89F1-768FC2964DBB

Fig. 16B Great roundleaf bat

##### Notes.

This species was observed in each of the transects in human-disturbed forest, national park forest, and human settlement areas located near forested areas. This species, like others in this genus, feeds primarily on flying insects inhabiting forested areas (Pavey 2021).

### ﻿Family Vespertilionidae

#### ﻿ Myotis horsfieldii

Taxon classificationAnimaliaChiropteraVespertilionidae

(Temminck, 1840)

6296BF0B-54AA-5101-8ADB-EF596E42B131

**Figure 17.**
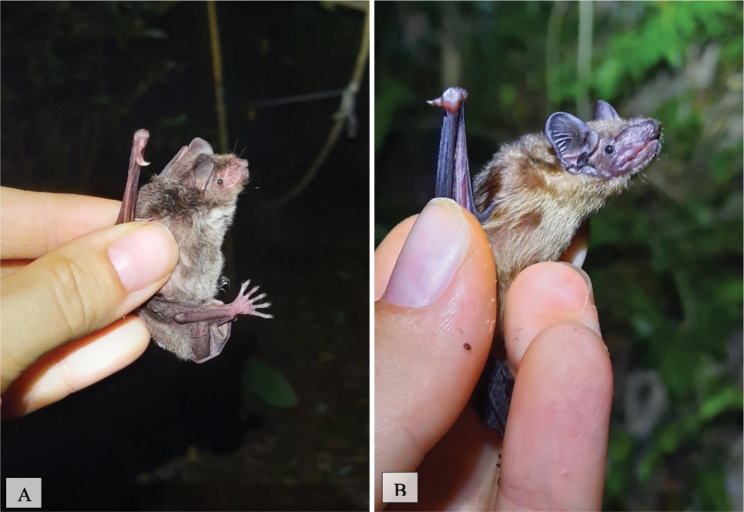
**A***Myotishorsfieldii***B***Tylonycterismalayana*.

Fig. 17A Himalayan whiskered bat

##### Notes.

This species was observed in human-disturbed forest, national park forest, and human habitat areas located near forested areas. This insectivorous species was observed throughout each of the mist net sites.

#### ﻿ Tylonycteris malayana

Taxon classificationAnimaliaChiropteraVespertilionidae

(Chasen, 1940)

E669AF4D-9117-5BFE-A4FF-51A1A7FC3AA4

Fig. 17B Bamboo bat

##### Notes.

This species, also insectivorous, was observed in human-disturbed forest, national park forest and human habitat areas located near forested areas. It was also caught at each mist net site throughout the island (Ruedi et al. 1990; Tu et al. 2017).

### ﻿Family Pteropodidae

#### ﻿ Pteropus hypomelanus

Taxon classificationAnimaliaChiropteraPteropodidae

(Temminck, 1853)

71665EE7-7D94-5C70-A1B7-0E53680D631B

##### Notes.

This species was observed in human settlement areas, human-disturbed forest areas, and national park forest areas. This species is fond of figs and is instrumental as a pollinator and seed disperser (Aziz et al. 2017).

#### ﻿ Cynopterus horsfieldii

Taxon classificationAnimaliaChiropteraPteropodidae

Gray, 1843

04C2EEF1-67D0-53CC-9E97-D4F13785AB0A

**Figure 18.**
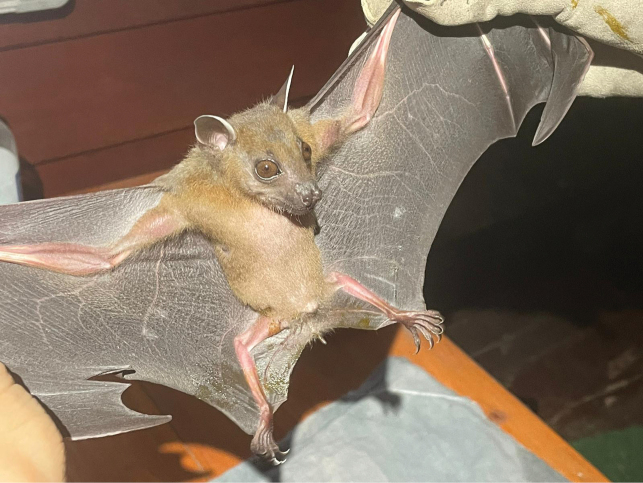
Cynopterushorsfieldii

Fig. 18 Horsfield’s short-nosed bat

##### Notes.

This fruit-eating species was trapped and observed in one human settlement area located near a forested area. This species can inhabit a variety of habitats from forest to agriculture and primarily consumes fruit and pollen (Campbell and Kunz 2006; Fletcher et al. 2012).

### ﻿Family Pteropodidae

#### ﻿ Cynopterus sphinx

Taxon classificationAnimaliaChiropteraPteropodidae

(Vahl, 1797)

D29D69BD-1208-5FD9-93C1-287CA81B5F79

**Figure 19.**
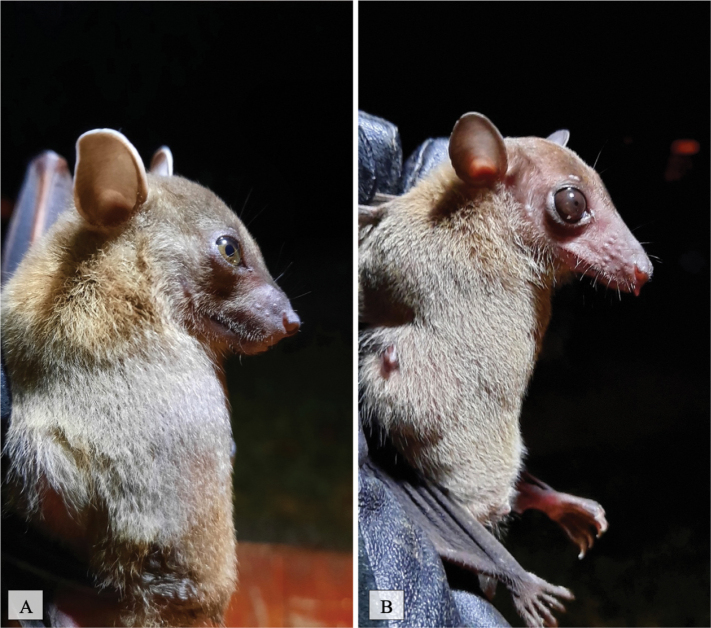
**A***Cynopterussphinx***B***Eonycterisspelaea*.

Fig. 19A Greater short-nosed fruit bat

##### Notes.

This fruit eating species (Bumrungsri et al. 2007) was observed in human-disturbed forest, national park forest, and human habitat areas located near forested areas. This species was trapped in mist net traps at all locations throughout the island.

#### ﻿ Eonycteris spelaea

Taxon classificationAnimaliaChiropteraPteropodidae

(Dobson, 1871)

1F5F9871-B175-592C-BD96-12A4231AFF96

Fig. 19B Dawn bat

##### Notes.

This species feeds on nectar and is an instrumental pollinator (Bumrungsri et al. 2013). It was observed in human-disturbed forest, national park forest, and human habitat areas located near forested areas. This species was caught at each mist net trap site throughout the island.

### ﻿Family Megadermatidae

#### ﻿ Megaderma spasma

Taxon classificationAnimaliaChiropteraMegadermatidae

(Linnaeus, 1758)

C138CFD0-998B-5E60-8D1D-30D88410C581

**Figure 20.**
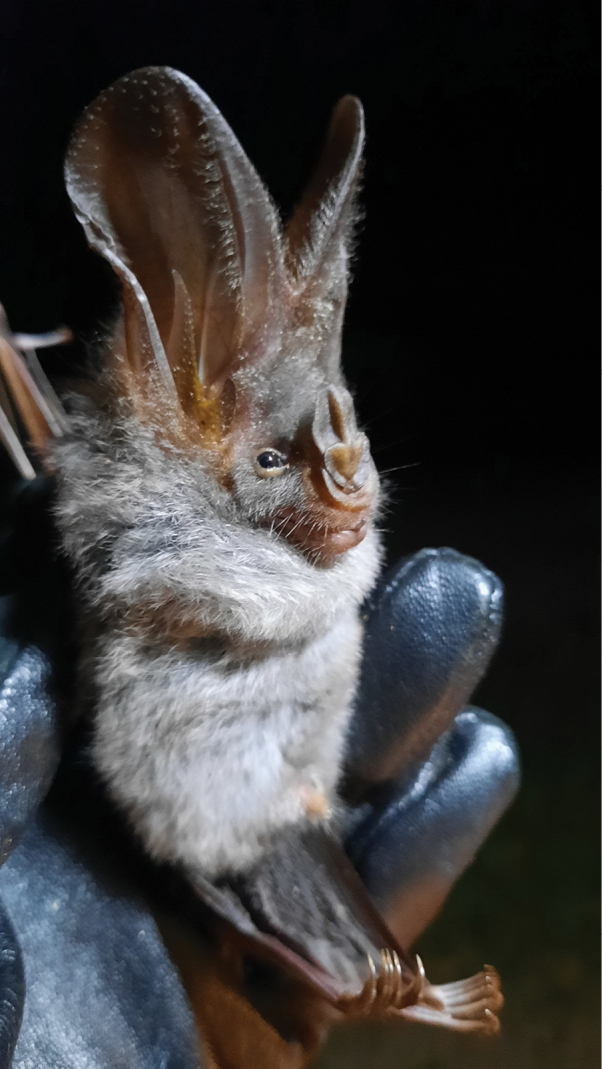
*Megadermaspasma* (lesser false vampire bat).

Fig. 20 Lesser false vampire

##### Notes.

This insectivorous (Balete 2010) species was observed in human-disturbed forest, national park forest, and human habitat areas located near forested areas. This species was caught on mist net traps in each of the trap sites throughout the island.

## ﻿Discussion

A total of 28 mammal species were detected throughout the island. One is critically endangered (*Manisjavanica*), one is endangered (*Nycticebuscoucang*), one vulnerable (*Rusaunicolor*), and two near threatened (*Ratufabicolor* and *Trachypithecusobscurus*). These critical findings underscore the need for robust conservation measures in the face of ongoing habitat loss and fragmentation. Unlike the mainland, an island habitat is finite and entirely restricted by water that allows no room for a population to shift ranges in response to environmental changes. Additionally, widespread species such as macaques and bats were observed across all surveyed habitats, suggesting their adaptability to varying environmental conditions on the island, showcasing the importance of adaptability for insular species (Guo et al. 2017).

The deployment of camera traps yielded valuable insights into the habits of elusive species like the *Paradoxurusmusangus* and *Manisjavanica*, which were seldom observed but captured on film in deep forest areas. However, challenges in species identification, particularly for bats and some rodents on camera traps and surveys, necessitated the use of live trapping and mist nets for accurate identification, indicating the mobility and wide habitat usage of these groups.

Each detection method had its strengths and weaknesses. Live traps and mist nets were the most effective way to capture and identify small mammals (rats and shrews) and bats. Live traps yielded eight species whereas mist nets yielded ten species. Camera traps yielded seven species while surveys yielded three species and opportunistic sightings yielded two different species sightings. The least intrusive method of detection, camera traps, were the most effective mode of detection when considering the total number of species detected despite identifiability (rats and bats that could not be accurately identified). The most intrusive method, surveys (human presence, torchlight, etc.) were the least effective method of detection. After camera traps, live traps were the most effective (and accurate) method of detection.

Due to the cryptic, nocturnal, or arboreal nature of animals such as the dusky leaf monkey, slow loris, or civet, a combination of detection methods was used. The slow loris and dusky leaf monkey are shy and primarily reside in the tree canopy making them difficult to detect via stationary camera traps placed just above ground level. The loris was only detected via night surveys due to their distinct eyeshine. The opportunistic sighting of the pangolin was pure luck; however, when using the camera trap: it was highly strategic as the first camera was placed in a location in the national park forest near a stream and a termite mound with damage. Each method had strengths and weaknesses, we simply capitalized on each of them.

Very little research has been conducted on the Island of Pha-ngan which is one explanation as to why this study adds 19 species more into the official national park list. In addition, during the Holocene period (more than 20 million years ago), Ko Pha-ngan was not isolated. Instead, it was an integral part of the Sunda Shelf, seamlessly connected to the landmasses that today constitute mainland Southeast Asia (Nutalaya et al. 1979). This historic connectivity implies that the island once harbored a more vibrant tapestry of biodiversity than is currently discernible. However, as sea levels rose and the process of insularization took hold, the island’s ecological richness started to wane. This phenomenon, where isolation typically results in diminished species diversity due to limited habitats and amplified exposure to disturbances, is consistent with established biogeography theories (MacArthur and Wilson 1967).

**Figure 21.**
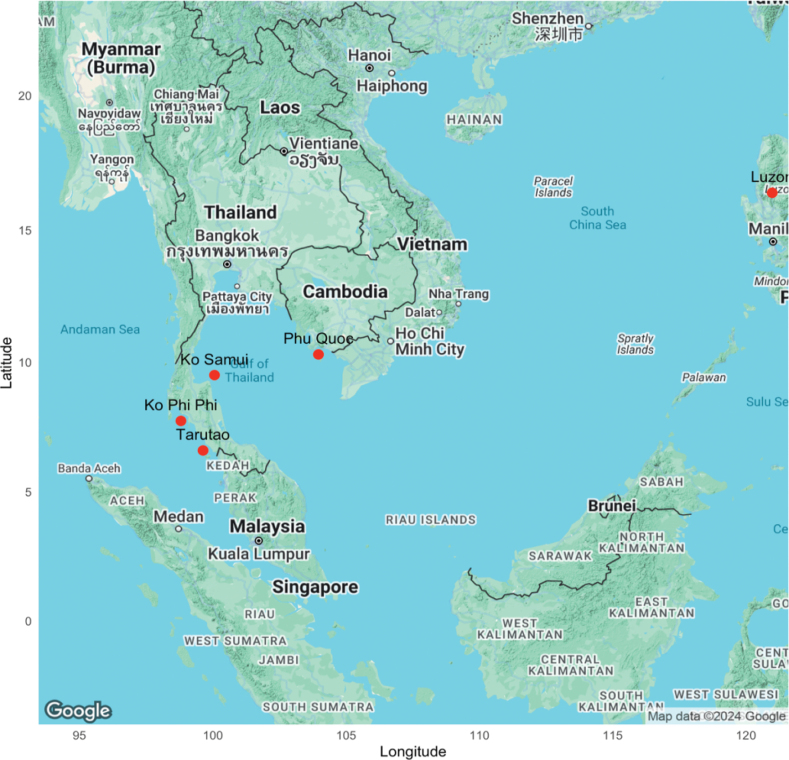
Map of all island areas mentioned in the discussion.

Similarly to Ko Pha-ngan, the nearby island of Samui, a mere 15 km from Ko Pha-ngan (Fig. 21) and a tourist hotspot, also lacks comprehensive documentation of its mammal populations, listing a mere 14 species from an old study (Marshall and Nongngork 1968). Similar to the records regarding Samui, there are also no known studies of the whole Surat Thani province; there are, however, numerous studies of protected areas within the province such as the Khao Sok National Park and the Khlong Saeng Wildlife Sanctuary that resides within the Khao Sok National Park area. Khao Sok (739 km^2^) is nearly six times the size of Pha-ngan island and ~ 11× the size of the Than Sadet-Ko Pha-ngan National Park (65 km^2^) itself. Within the Khao Sok area, there resides ~ 111 mammals (Thai National Parks 2024a), more than 5× the number of species on the island. The Khao Sok expanse is large enough to support a biodiverse ecosystem consisting of large mammals such as fishing cats, bears, and elephants. Ko Pha-ngan does not have the quantity or area of habitat to support such large mammals; however, the subset of species found on the island compared to the mainland areas are representative of insularization and island biogeography.

Across the mainland, on the Andaman side of the peninsula, lies Tarutao Island, a mere 26 km off the mainland (Fig. 20). A UNESCO heritage site and national park, Tarutao harbors ~ 30 mammal species (National Parks of Thailand 2024) as well as 19 species of bat (Bumrungsri et al. 2010). In contrast to Ko Pha-ngan, Tarutao, with its pristine forested and limestone habitat, experiences minimal human disturbance and boasts a higher average of mammalian diversity inhabiting its 152 km^2^. Common species such as *Callosciuruscaniceps* (grey-bellied squirrel), *Tupaiabelangeri* (common shrew), and *Macacafascicularis* (macaque) are found on both islands and throughout the mainland; however, species such as *Prionailurusviverrinus* (fishing cat), and *Galeopterusvariegatus* (flying lemur) are only found on Tarutao, probably due to the presence of each species’ preferred habitat (Thai National Parks 2024b). The importance of a healthy ecosystem, marked by a balance of species, is evident; disturbances, natural or human-induced, can significantly alter this balance which is visible on Ko Pha-ngan with its unfortunate abundance of human disturbance.

North of Tarutao in the Andaman Sea is also the Phi Phi Archipelago (Fig. 20). Ko Phi Phi Don and Ko Phi Phi Leh are the two largest (9.408 km^2^ and 1.8 km^2^, respectively) of the six-island complex. Also popular tourist destinations, the islands support eight mammals (Nadee 1993) with only one species (*Callosciuruscaniceps*) in common with Ko Pha-ngan. The natural steep karst formations coupled with small island size and anthropogenic changes account for the limited capacity for mammalian diversity in addition to the lack of recent data.

Unlike the limited island area of the Ko Phi Phi Archipelago, other islands in Southeast Asia such as the very large Luzon Island (109,964 km^2^) in the Philippines, can support greater mammal diversity. Luzon, rich in biodiversity, boasts 56 non-flying mammal species (Heaney et al. 2016), compared to Ko Pha-ngan’s more modest count of 17 non-flying mammals. Factors such as Luzon’s larger array of habitats which include unique montane forests home to multiple endemic rodent species, lower levels of historical deforestation, and stronger conservation initiatives contribute to this difference. Luzon has been extensively studied whereas Ko Pha-ngan is at the beginning stages of research, and the potential for additional species for Ko Pha-ngan is high. This underscores the potential to enhance biodiversity on Ko Pha-ngan through focused habitat preservation, restoration, and additional research.

Distance from the mainland is the classic biogeographical explanation for the varying mammal populations on the islands; however, as the animals are forced to survive in restricted habitat, they occupy a niche maximizing their chance of survival as their habitat changes (Podchong et al. 2009). For instance, Phu Quoc Island in Vietnam, despite being the largest Vietnamese island and close to the mainland, only boasts 17 mammalian species, which contradicts existing theories (Abramov et al. 2007). This variation highlights the importance of localized conservation efforts tailored to each island’s historical and ecological context.

The landscape of Ko Pha-ngan, shaped by human activities such as tin mining in the 1970s and the development of coconut plantations, faces new threats from burgeoning tourism. These human-driven changes have significant adverse effects on ecosystems, especially on island habitats as modifications to the landscape are the primary factor driving changing biodiversity (Gössling 2002: Graham et al. 2017). This is potentially detrimental for reclusive species primarily found in national park and human-disturbed forest habitats such as *Nycticebuscoucang*, *Ratufabicolor*, and *Trachypithecusobscurus* as their living space continues to shrink. Being arboreal creatures they may become more vulnerable to habitat fragmentation as they are dependent on canopy cover and not as well adapted to human-built structures. As tourism increases and the demand for resorts, roads, and recreational areas grows, the remaining forests become more isolated, limiting the animals’ ability to move between feeding, breeding, and sheltering areas (Berger-Tal and Saltz 2019).

The presence of human settlements and tourist resorts on the island raises concern for potential opportunistic hunting, especially for species that are thought to be or perceived as pests or have commercial value. Bats, such as *Pteropushypomelanus* have historically been hunted for food throughout Southeast Asia making monitoring populations the very least that can be done in an effort to mitigate such a practice. While encroachment continues, conflicts between humans and wildlife may increase (Mildenstein et al. 2016).

The influx of tourists to Ko Pha-ngan puts pressure on native habitats such as national park forest and human-disturbed forest areas. While small islands cover only a fragment of global landmass, they unfortunately hold one third of the world’s threatened species (Deidun 2011). Recognizing the risks to the island’s fragile biodiversity is crucial because economic growth and ecological changes often go hand in hand (Russell and Kueffer 2019). Human disruptions affect island species, particularly those in specific environmental niches such as arboreal species or those moving through areas that were forested and are no longer can be impacted greatly by habitat fragmentation (Berger-Tal and Saltz 2019). As the tourist industry on the island grows the demand for development increases. This only perpetuates the fragmentation of habitat (Graham et al. 2017) negatively impacting the wildlife on the island trapping vulnerable species such as the loris or dusky leaf monkey to their ever-shrinking niches in the island’s ecosystem.

## ﻿Conclusion

Our study has illuminated the presence of 28 mammalian species on Ko Pha-ngan, including critically endangered (*Manisjavanica*, pangolin) and endangered (*Nycticebuscoucang*, slow loris) species whose conservation is now paramount. Prioritizing the protection of all threatened tax not only the critically endangered and endangered species, but also, the near threatened Pteropuscf.hypomelanus (island flying fox), *Trachypithecusobscurus* (dusky leaf monkey), and *Ratufabicolor* (giant black squirrel) as well as the vulnerable *Rusaunicolor* (sambar deer) ensures a more biodiverse island. The results underscore the intricate balance between human activity and wildlife in small island ecosystems. In addition, they call attention to the importance of national park forest areas for *Nycticebuscoucang*, and *Ratufabicolor*. The relative scarcity of these species in human-disturbed forest and human settlement areas suggest that habitat degradation poses a significant threat to their populations. Conservation strategies should focus on preserving national park forest areas and limiting further encroachment into these critical habitats.

## Supplementary Material

XML Treatment for Paradoxurus musangus

XML Treatment for Herpestes javanicus

XML Treatment for Rusa unicolor

XML Treatment for Sus scrofa

XML Treatment for Callosciurus erythraeus

XML Treatment for Callosciurus caniceps

XML Treatment for Ratufa bicolor

XML Treatment for Maxomys surifer

XML Treatment for Rattus andamanensis

XML Treatment for Rattus exulans

XML Treatment for Rattus tanezumi

XML Treatment for Crocidura fuliginosa

XML Treatment for Tupaia belangeri

XML Treatment for Macaca fascicularis

XML Treatment for Trachypithecus obscurus

XML Treatment for Nycticebus coucang

XML Treatment for Manis javanica

XML Treatment for Rhinolophus affinis

XML Treatment for Rhinolophus pusillus

XML Treatment for Hipposideros larvatus

XML Treatment for Hipposideros armiger

XML Treatment for Myotis horsfieldii

XML Treatment for Tylonycteris malayana

XML Treatment for Pteropus hypomelanus

XML Treatment for Cynopterus horsfieldii

XML Treatment for Cynopterus sphinx

XML Treatment for Eonycteris spelaea

XML Treatment for Megaderma spasma

## ﻿Additional information

### Author ORCIDs

Dawn R. Cook-Price https://orcid.org/0009-0000-5035-9642

Olga N. Petko https://orcid.org/0009-0004-5131-0145

Sunchai Makchai https://orcid.org/0009-0008-4067-7442

Taksin Artchawakom https://orcid.org/0000-0003-3004-6539

Pongthep Suwanwaree https://orcid.org/0000-0002-3047-2717

### Data availability

All of the data that support the findings of this study are available in the main text.
